# DHRS2 mediates cell growth inhibition induced by Trichothecin in nasopharyngeal carcinoma

**DOI:** 10.1186/s13046-019-1301-1

**Published:** 2019-07-10

**Authors:** Xiangjian Luo, Namei Li, Xu Zhao, Chaoliang Liao, Runxin Ye, Can Cheng, Zhijie Xu, Jing Quan, Jikai Liu, Ya Cao

**Affiliations:** 1 Key Laboratory of Carcinogenesis and Invasion, Chinese Ministry of Education, Department of Radiology, Xiangya Hospital, Central South University, Changsha, Hunan 410078 People’s Republic of China; 2 Cancer Research Institute, School of Basic Medicine, Central South University, Changsha, Hunan 410078 People’s Republic of China; 3 Key Laboratory of Carcinogenesis, Chinese Ministry of Health, Changsha, 410078 Hunan China; 4 Molecular Imaging Research Center of Central South University, Changsha, 410078 Hunan China; 5 Department of Pathology, Xiangya Hospital, Central South University, Changsha, 410078 Hunan China; 6 School of Pharmacy, South-central University for Nationalities, Wuhan, 430074 Hubei China

**Keywords:** DHRS2, Trichothecin, Cell cycle, Lipid metabolism, Nasopharyngeal carcinoma

## Abstract

**Background:**

Cancer is fundamentally a deregulation of cell growth and proliferation. Cancer cells often have perturbed metabolism that leads to the alteration of metabolic intermediates. Dehydrogenase/reductase member 2 (DHRS2) belongs to short-chain alcohol dehydrogenase/reductase (SDR) superfamily, which is functionally involved in a number of intermediary metabolic processes and in the metabolism of lipid signaling molecules. DHRS2 displays closely association with the inhibition of cell proliferation, migration and quiescence in cancers.

**Methods:**

3-(4,5-dimethylthiazol-2-yl)-5-(3-carboxymethoxyphenyl)-2-(4- sulfophenyl)-2H-tetrazolium (MTS), 5-ethynyl-2′-deoxyuridine (EdU) and colony formation assays were applied to evaluate the proliferative ability of nasopharyngeal carcinoma (NPC) cells. We performed lipid metabolite profiling using gas chromatography coupled with mass spectrometry (GC/MS) to identify the proximal metabolite changes linked to *DHRS2* overexpression. RNA sequencing technique combined with differentially expressed genes analysis was applied to identify the expression of genes responsible for the anti-tumor effect of trichothecin (TCN), a natural sesquiterpenoid compound isolated from an endophytic fungus.

**Results:**

Our current findings reveal that DHRS2 affects lipid metabolite profiling to induce cell cycle arrest and growth inhibition in NPC cells. Furthermore, we demonstrate that TCN is able to induce growth inhibition of NPC in vitro and in vivo by up-regulating DHRS2.

**Conclusions:**

Our report suggests that activating DHRS2 to reprogram lipid homeostasis may be a target for the development of targeted therapies against NPC. Moreover, TCN could be exploited for therapeutic gain against NPC by targeting DHRS2 and it may also be developed as a tool to enhance understanding the biological function of DHRS2.

**Electronic supplementary material:**

The online version of this article (10.1186/s13046-019-1301-1) contains supplementary material, which is available to authorized users.

## Background

Cancer is fundamentally a deregulation of cell growth and proliferation [1]. Cancer cells often have perturbed metabolism that leads to the alteration of metabolic intermediates [2–6]. In addition to glucose metabolism, lipid metabolism is also perturbed in rapidly proliferating cells [7–9]. Lipid metabolites play central roles in intermediary metabolism, in that they not only provide substrates for biological processes, but also impact on the control of cell cycle, proliferation and apoptosis [10–15]. Sphingosine-1-phosphate (S1P), a bioactive lipid, is reported to protect oral squamous cell carcinoma cells from cisplatin-induced death [16, 17]. Addition of unsaturated fatty acids (FAs) attenuates betulinic acid-induced cardiolipin saturation and subsequent cytotoxicity in HeLa cells [14]. Whereas, saturated FAs palmitate and stearate can substantially inhibit the growth of pancreatic cancer cells [13].

Short-chain alcohol dehydrogenase/reductase (SDR) superfamily includes NAD/NADP- dependent oxidoreductases, which are functionally involved in a number of intermediary metabolic processes and in the metabolism of lipid signaling molecules [18]. Dehydrogenase/reductase member 2 (DHRS2) is a member of the SDR superfamily with the nomenclature name SDR25C1 (also known as HEP27). *DHRS2* gene is localized in chromosome 14q11.2, a region with high-frequency heterozygous loss in many tumors, including nasopharyngeal carcinoma [19], gastrointestinal stromal tumors [20], mesothelioma [21], esophageal squamous cell carcinoma [22] and metastatic lung adenocarcinomas [23]. This supports the notion that DHRS2 might play functional roles in tumorigenesis and malignant progression.

DHRS2 is originally cloned from hepatocellular carcinoma cells (HepG2) and associates closely with the inhibition of cell proliferation, migration and quiescence [22, 24–26]. In HepG2 cells, DHRS2 is up-regulated accompanied by cell G1 phase arrest induced by the treatment of sodium butyrate, a histone deacetylase inhibitor [27]. DHRS2 can act as negative regulator of murine double minute 2 (MDM2), subsequently promote p53 stabilization and accumulation [24]. Down-regulation of DHRS2 contributes to gastric carcinogenesis through interacting with MDM2 and confers insensitivity to 5-FU therapy through a p53-dependent pathway [25].

Nasopharyngeal carcinoma (NPC) is one of the major subtypes of head and neck cancers and arises from the epithelial cells of the nasopharynx. NPC represents a serious health problem in Southern China and Southeast Asia. Due to the secluded structure and intrinsic invasiveness of the disease, majority of NPC patients are diagnosed with advanced diseases (stages III and IV) and poor outcome. So far, no effective targeted therapy for advanced NPC is available [28–31].

Natural products provide unique source for the discovery of innovative drugs that rationally target the aberrant molecular signaling leading to cancer [32–37]. Trichothecin (TCN) is a secondary fungal metabolite isolated from an endophytic fungus of the herbal plant Maytenus hookeri Loes [38]. Although previous studies have shown that TCN exerts anti-tumor activity by induction of cell apoptosis [39, 40], the underlying mechanisms are not completely understood.

In this manuscript, we assess the role of metabolic enzyme DHRS2 in cell cycle arrest and growth inhibition of NPC cells and the underlying mechanism is further explored. Moreover, the effect of TCN on growth inhibition through up-regulation of DHRS2 is investigated. The present study aims to establish a mechanistic connection between metabolism rewiring and alteration of cell cycle and proliferation in cancer cells, and further develop novel pharmacological tools against NPC by induction of DHRS2.

## Materials and methods

### Cell culture

The immortalized nasopharyngeal epithelial NP69 cell line was cultured in keratinocyte serum-free medium (SFM) (Invitrogen, Carlsbad, USA) with the SFM-Growth supplement. The immortalized nasopharyngeal epithelial NP460 cell line was grown in a 1:1 ratio of Defined Keratinocyte-SFM (Gibco, NY, USA) supplemented with growth factors. The human NPC cell lines, including HK1, C666–1, CNE1, CNE2, CNE1-LMP1, HNE1, HNE2, HONE1, 5–8F, 6-10B, SUNE1 and lung carcinoma cell lines, including A549 (ATCC No.CCL-185) and H1299 (ATCC No.CRL-5803) cell lines were grown in RPMI 1640 media supplemented with 10% v/v heat-inactivated fetal bovine serum (FBS), 1% w/v glutamine and 1% w/v antibiotics. All the cell lines involved were cultured at 37 °C in a humidified incubator containing 5% CO_2_.

### Reagents, chemicals and plamids

TCN was provided by Kunming Institute of Botany, the Chinese Academy of Sciences (purity > 99%, HPLC analysis) as previously described [41] (Fig. 4a). Dimethyl sulphoxide (DMSO, Sigma) was used to dissolve TCN. The final concentration of DMSO in the culture media was kept less than 0.1% v/v which had no significant effect on the cell growth. Elaidic acid and Oleic acid were purchased from MedChemExpress (NJ, USA).

The antibodies against β-actin and CDK4 were purchased from Santa Cruz Biotechnology (Santa Cruz, CA, USA). The antibodies against RB, pRB and Ki67 were obtained from Cell Signaling Technologies (Danvers, MA, USA). The antibody to detect DHRS2 (PA5–25258) was from Thermo Fisher Scientific. Anti-cyclin D1 was from Bioworld Technology.

pEZ-Lv105-DHRS2 was from GeneCopeia. The open reading frame of *DHRS2* gene was inserted into the lentivirus vector pEZ-Lv105 using Gateway® recombination technology.

### Cell proliferation assays

For EdU assay, the cell proliferation rate was assessed by plating cells into 96-well plates with 2 × 10^3^ cells per well and the Integrated Optical Density (IOD) was measured by Operetta CLS (Perkin Elmer, USA) with BeyoClick™ EdU Kit (Beyotime, China). The data were analyzed by Harmony 4.5 software.

For BrdU assay, the cell proliferation rate was evaluated by plating cells into 6-well plates with 2 × 10^5^ cells per well and the fluorescence intensity was measured by flow cytometry (Becton–Dickinson) with BrdU Staining Kit (eBioscience, USA).

### Colony formation

Cells were plated into 6-well plates with 1 × 10^3^ cells per well in triplicate. After 10–14 days, cells were washed with 1 × PBS, fixed in methanol for 20 min and stained with crystal violet for 15 min at room temperature. Colonies containing more than 50 cells were counted using the Image J software.

### Cell cycle analysis

Flow cytometry was used to quantitatively detect the cell-cycle distribution. Cells (2 × 10^6^) were fixed in pre-cooled 75% ethanol, incubated with propidium iodide (Sigma-Aldrich, MO, USA). Flow cytometry (Becton–Dickinson, USA) was used to analyze DNA content. Cell cycle analysis was performed by flowjo 7.6 software.

### GC/MS measurements

Cells were washed thrice by pre-cooled phosphate-buffered saline and fixed with methanol, put periodically twice from − 80 °C for 30 min to room temperature for 15 min, harvested by cell scraper and stored at − 80 °C until the analysis was conducted. FAs metabolites were measured essentially as described previously [42]. The relative abundances of the species in the sample-extracts was determined using a gas chromatography system coupled to a mass spectrometer (GC–MS-QP2010 Ultra, Shimadzu, Kyoto, Japan). Heptadecanoic acid was added as an internal standard and thirty-seven mixed Fatty Acid Reference Standard (FAMQ-005, AccuStandard, New Haven, USA) as an external standard. The thirty-seven metabolites were identified by their corresponding chemical standards. Data were analyzed using GCMSsolution software Ver.4.

### RNA sequencing and screening of DEGs

The mRNA obtained from ∼10 μg of total RNA was isolated, fragmented, converted to cDNA, and amplified by PCR. The double stranded PCR products were heat denatured and circularized. The single strand circle DNAs (ssCir DNAs) were formatted as the final library. The library was validated on the Agilent Technologies 2100 bioanalyzer and sequenced using BGISEQ-500 (BGI, Shenzhen, China).

The data analysis screened the DEGs among the treatments and performed a Gene Ontology (GO) functional enrichment analysis and Kyoto Encyclopedia of Genes and Genomes (KEGG) pathway enrichment analysis of the DEGs. We applied the NOIseq method [43] to screen the DEGs between two groups. Genes were deemed significantly differentially expressed at a probability of≥0.8 and an absolute value of log_2_ ratio ≥ 1 (the difference in expression was greater than 2). The Blast2GO program with default parameters was used to perform the GO functional enrichment analysis; after obtaining the GO annotations for the DEGs, the WEGO software was used to perform the GO functional classification of the DEGs. The results of the GO functional classification are displayed in three domains: Biological processes, cellular components, and molecular functions [44]. In addition, the biological processes in which the DEGs were involved were defined by assigning the DEGs to metabolic pathways or signal transduction pathways using the KEGG annotation [45]. For the GO terms, we used corrected *P*-values < 0.05 to demonstrate a significant enrichment of the gene sets. KEGG pathways with threshold Q-values≤0.25 were considered significantly enriched in the DEGs.

### Immunohistochemical analysis

The tumor tissue sections were deparaffinized in environmentally friendly dewaxing agent (solarbio, china) and rehydrated with an ethanol-aqueous solutions of decreasing concentrations. For antigen retrieval, tissue sections were incubated in 10 mM sodium citrate buffer (pH = 6.0) for 20 min in a microwave oven. The endogenous peroxidase activity was removed by incubating with 3% hydrogen peroxide for 10 min and was blocked in normal donkey serum for 30 min. The primary antibodies (anti-DHRS2, anti-Ki67) were applied at 4 °C overnight. Chromogen was developed using DAB (Zsgb-bio, China) and counterstained with hematoxylin staining kit. Immunohistochemical staining of these sections was evaluated based on all of the available tumor cells or epithelial cells meeting the typical morphological criteria by 3 pathologists using the qualitative scale that is described in the literature. The number of cells stained was scored as 0 (no staining), 1 (< 1/3 positive cells), 2 (> 1/3 and < 2/3 positive cells) and 3 (> 2/3 positive cells). The intensity of staining ranged from 1 (weak) to 3 (strong). The immune reactive score was calculated by multiplying the percentage of positive cells and staining intensity.

### Tumor xenograft studies

To assess the role of DHRS2 in NPC tumors in vivo, female BALB/c nu/nu mice (5 animals per group, 5 weeks old) were subcutaneously inoculated with HK1-CON or HK1-DHRS2 cells (3 × 10^6^).

To investigate the inhibitory effect of TCN on NPC in vivo, xenograft experiment using HK1 cells was performed by subcutaneous injection of 3 × 10^6^ cells into 5-week-old BALB/c nu/nu female mice. After the tumors grew to a volume of about 80–100 mm^3^, mice were randomly divided into three groups (*n* = 6 for each): untreated, treatment with TCN (1 mg/kg) or DDP (5 mg/kg) by intraperitoneal injection every other day for 5 weeks, respectively.

In both experiments, tumor volume was calculated according to the formula (V = length x width^2^/2). At the end of experiments, the mice were euthanized by CO_**2**_ inhalation and the tumors were stripped and weighed. Animal care experimental procedures were conducted in accordance with the approval of Xiangya hospital of Central South University (Changsha, China).

### Statistical analysis

All statistical calculations were performed with the statistical software program SPSS ver.16.0. Differences between various groups were evaluated by a two-tailed Student’s t test and a *p* value < 0.05 was considered statistically significant.

## Results

### DHRS2 inhibits cell growth in nasopharyngeal carcinoma cells

**Fig. 1:**
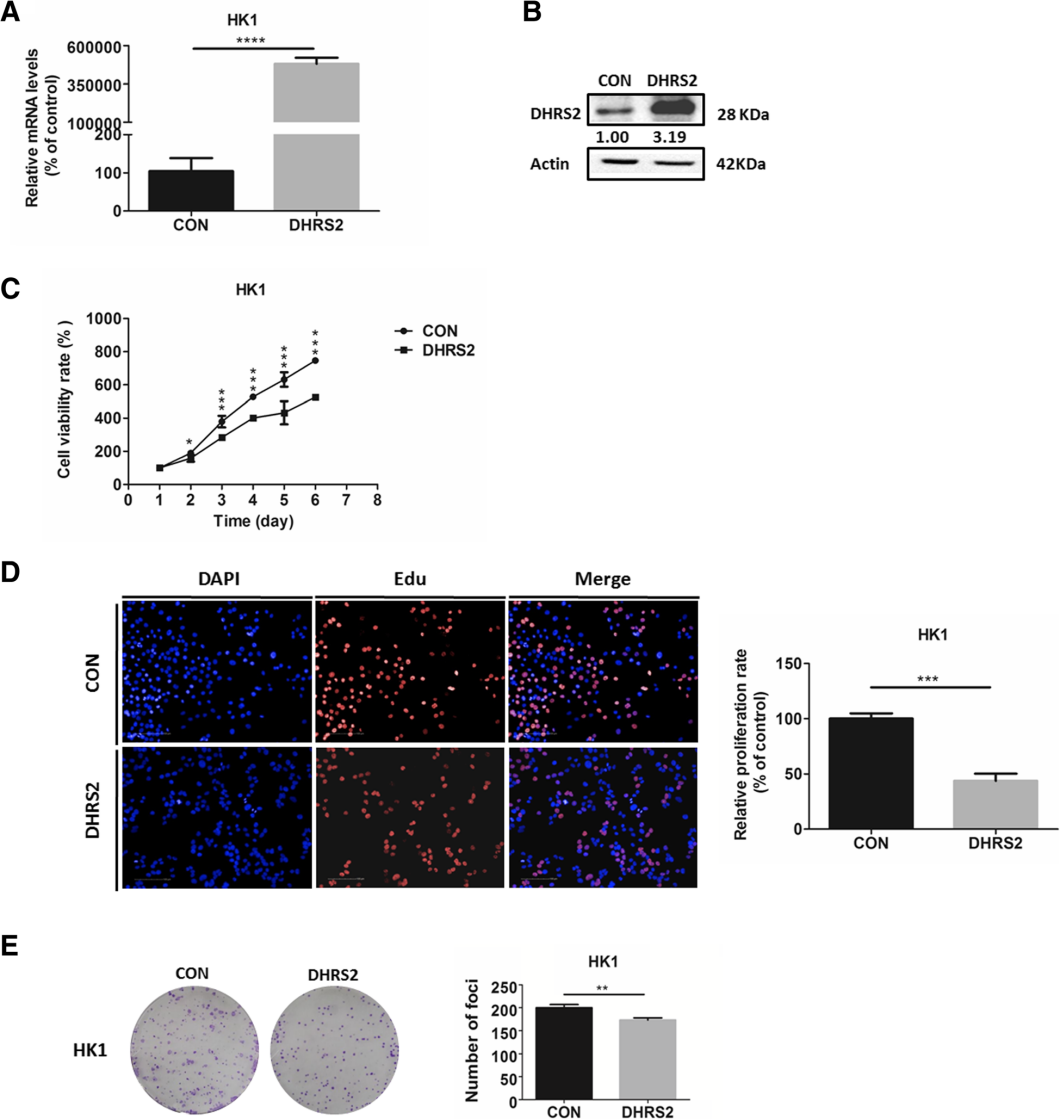
DHRS2 inhibits cell proliferation in NPC cells. The mRNA (**a**) and protein (**b**) levels of DHRS2 in HK1-CON and HK1-DHRS2 cells. **c** Cell growth of HK1-CON and HK1-DHRS2 cells over a 6-day period was analyzed by MTS assay. **d** HK1-CON and HK1-DHRS2 cells were seeded on a 96-well plate overnight and the EdU-positive proliferative cells (red) were determined by Operetta CLS high-screening imaging system, when nuclei were stained blue. The ratio of EdU-positive cells was calculated by Harmony 4.5 and shown in bar graphs. **e** The foci formation ability of HK1-CON and HK1-DHRS2 cells was analyzed by colony formation assay. Data are shown as mean values ± S.D. of independent, triplicate experiments. The asterisks (*,**,***) indicate significant differences (*p* < 0.05, *p* < 0.01, *p* < 0.001,respectively)

To gain insight into the role of DHRS2 in NPC, first we examined the protein levels of DHRS2 in multiple NPC cells. We found much lower expression of DHRS2 in NPC cells, especially in HK1, relative to the immortalized nasopharyngeal epithelial cells, NP69 and NP460 (Additional file 1: Figure S1A). Next, we established stable HK1-DHRS2 cell line by over-expressing DHRS2 into HK1 cells and the control HK1-CON cells (Fig. 1a-b). Cell growth was markedly suppressed in HK1-DHRS2 cells compared to that in HK1-CON cells (Fig. 1c). EdU (5-ethynyl-2′-deoxyuridine) assay was further performed to evaluate the cell proliferative ability. As a thymidine mimic, EdU can enter into DNA double-strands during the DNA-synthetic S phase. Compared to the control cells, the ratio of EdU-positive cells was markedly decreased in HK1-DHRS2 cells (Fig. 1d). Moreover, over-expression of *DHRS2* significantly reduced the foci formation in HK1 cells (Fig. 1e). Nevertheless, it should be noted that using single clone of HK1-CON and HK1-DHRS2 is the limitation of this study. The comparison of different stable clones helps to exclude that some specific clone shows decreased growth for other reasons.

**Fig. 2:**
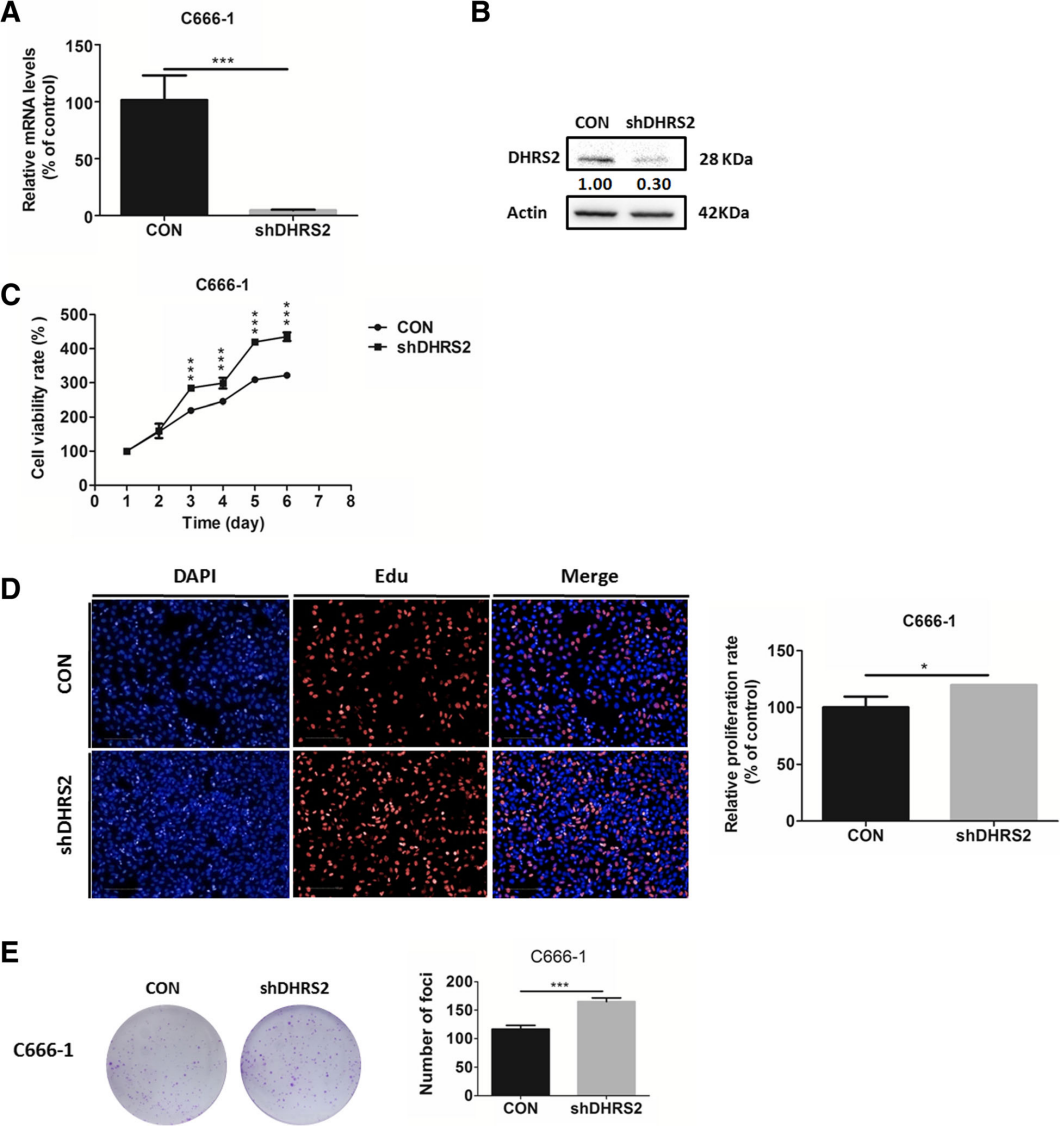
DHRS2 knockdown promotes cell proliferation in NPC cells. The mRNA (**a**) and protein (**b**) levels of DHRS2 in C666–1-CON and C666–1-shDHRS2 cells. **c** Cell growth of C666–1-CON and C666–1-shDHRS2 cells over a 6-day period was analyzed by MTS assay. **d** C666–1-CON and C666–1-shDHRS2 cells were seeded on a 96-well plate overnight and the EdU-positive proliferative cells (red) were determined by Operetta CLS high-screening imaging system, when nuclei were stained blue. The ratio of EdU-positive cells was calculated by Harmony 4.5 and shown in bar graphs. **e** The foci formation ability of C666–1-CON and C666–1-shDHRS2 cells was analyzed by colony formation assay. Data are shown as mean values ± S.D. of independent, triplicate experiments. The asterisks (*,**,***) indicate significant differences (*p* < 0.05, *p* < 0.01, *p* < 0.001,respectively)

Furthermore, short hairpin RNA (shRNA)(1#,2#,3#) targeting *DHRS2* was transfected into C666–1 cells, respectively. We confirmed that shDHRS2 3# exhibited the best knock down efficiency of *DHRS2* (Additional file 1: Figure S1B, Fig. 2a-b). Accordingly, we employed C666–1 cell line stably transfected with shDHRS2 3# (designated as shDHRS2) for further investigation. We observed that knockdown of DHRS2 promoted cell growth (Fig. 2c) and enhanced the ratio of EdU-positive cells (Fig. 2d). Colony formation assay further confirmed the conclusion (Fig. 2e).

**Fig. 3:**
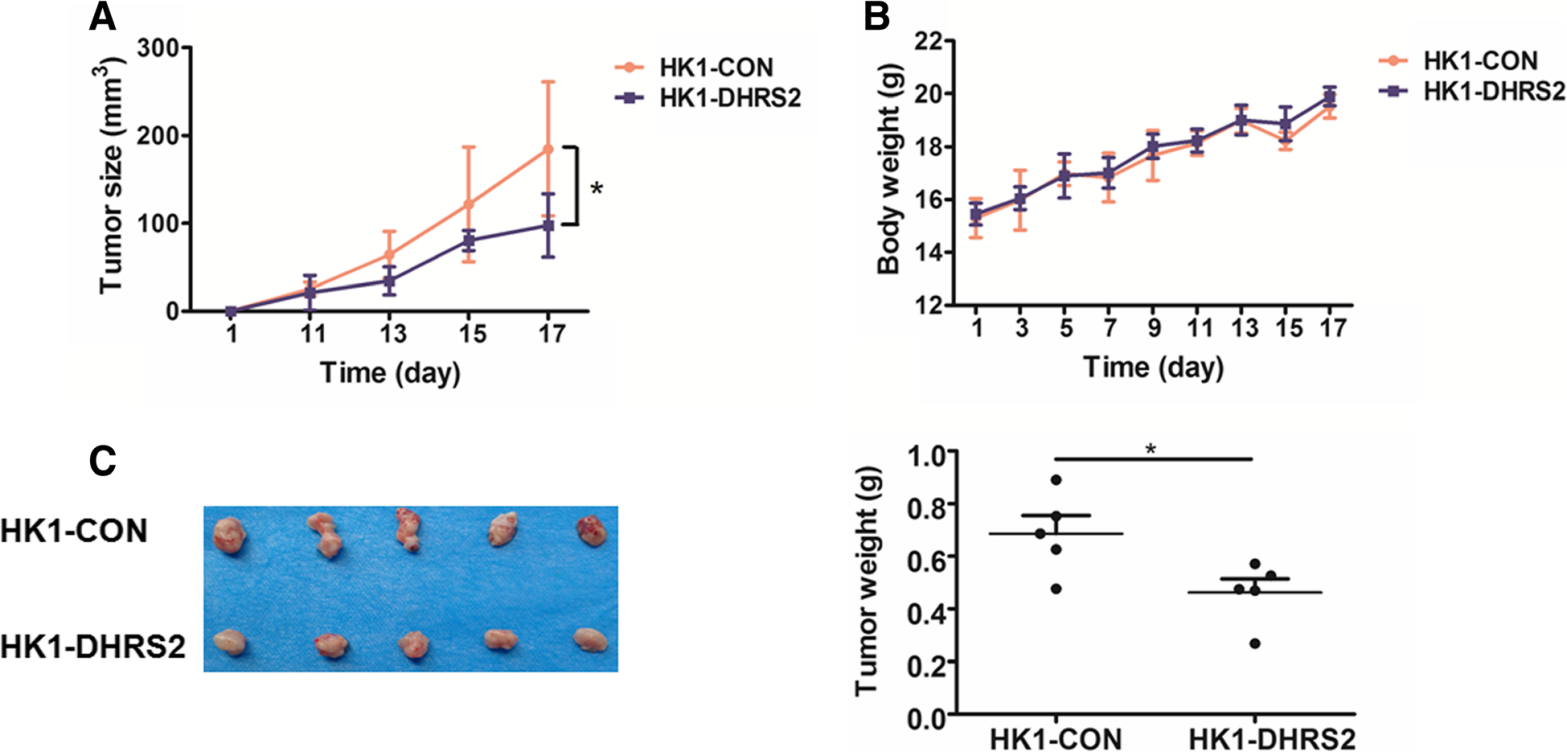
DHRS2 Overexpression inhibits NPC growth in vivo. **a** Growth curve of HK1-CON and HK1-DHRS2 cells in vivo. Female BALB/c nu/nu mice were subcutaneously inoculated with HK1-CON or HK1-DHRS2 cells (*n* = 5 per group). Tumor volume was examined every other day and shown in the graph. **b** During the experiment, body weight of the mice in each group was monitored and shown in the graph. **c** At the end of the experiment, the mice were sacrificed and the tumors were separated. Tumor mass of each group was weighed and shown in the graph

To assess the role of DHRS2 in NPC tumors in vivo, stable HK1-CON and HK1-DHRS2 cells were used to generate xenograft tumor models. We observed that mice injected with HK1-DHRS2 cells displayed delayed tumor occurrence. At the 11st day after injection, the ratios of mice with detectable tumor are 5/5 and 2/5, respectively for HK1-CON and HK1-DHRS2 groups (Fig. 3a). Overexpression of DHRS2 resulted in reduction in tumor size and tumor mass, with no appreciable effect on mice body weight (Fig. 3b-c).

Taken together, these results indicate that DHRS2 inhibits NPC cell proliferation both in vitro and in vivo.

### DHRS2 affects lipid metabolite profiling to induce cell cycle arrest in NPC cells

**Fig. 4:**
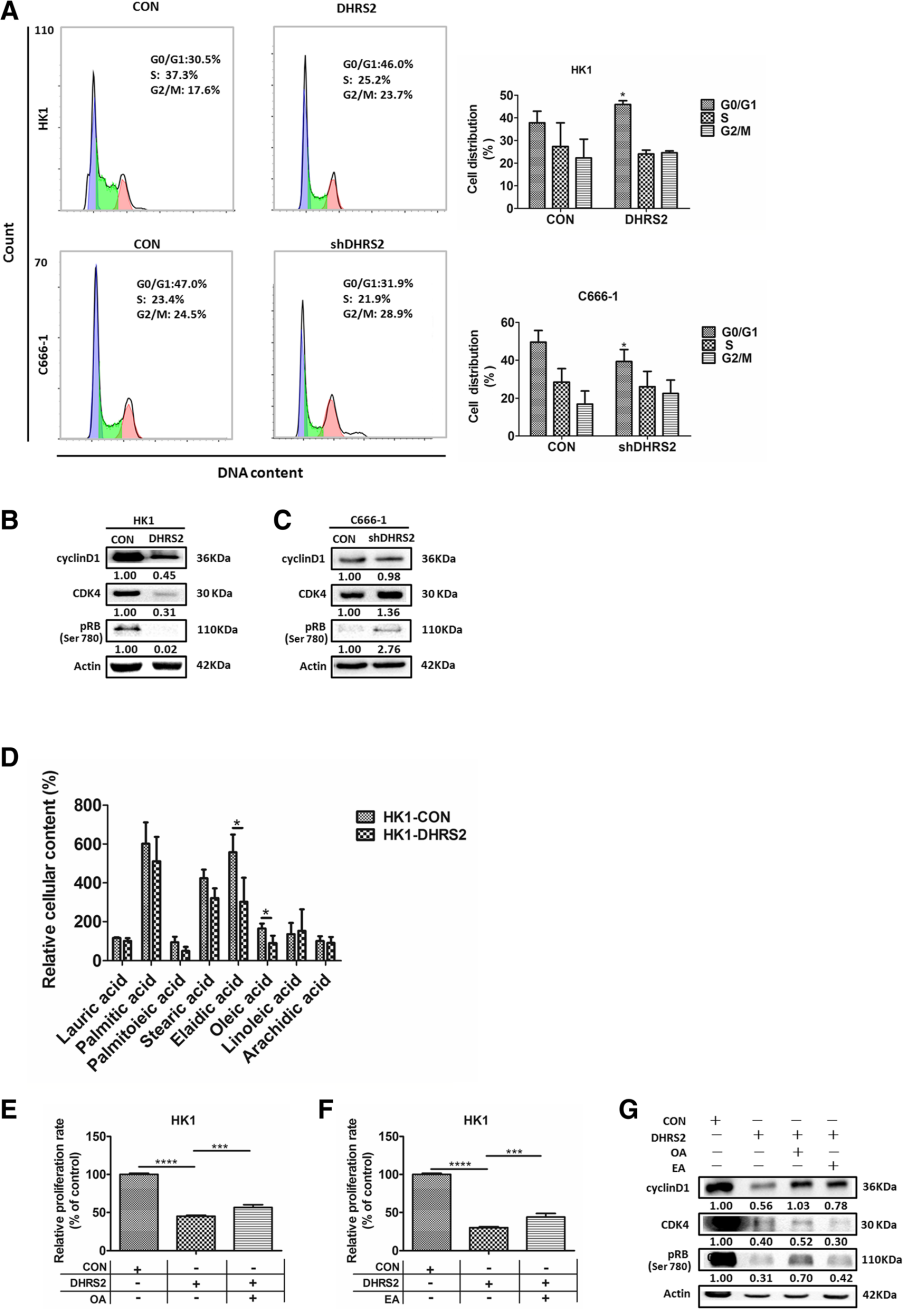
DHRS2 alters lipid profiling to induce cell cycle arrest in NPC cells. **a** The cell distribution was determined by flow cytometry in HK1-DHRS2 and C666–1- shDHRS2 cells compared with each vector control cells. The protein levels of cyclinD1, CDK4 and pRB (Ser780) were detected by western blot in HK1-CON / HK1-DHRS2 (**b**) and C666–1-CON / C666–1-shDHRS2 (**c**) cells. β-actin was used as a loading control. **d** The relative cellular content of FAs in HK1-CON / HK1-DHRS2 cells was analyzed by GC-MS. **e**-**f** Cell proliferation rate of each designated group was analyzed by MTS assay. After 16 h of starvation, HK1-CON and HK1-DHRS2 cells were treated in the absence or presence of 62.5uM OA or 62.5uM EA for 72 h as designated in each group and applied for MTS assay. **g** The protein levels of cyclinD1, CDK4 and pRB (Ser780) were detected by western blot assay. HK1-CON and HK1-DHRS2 cells were treated as (**e**-**f**) and applied for western blot. Data are shown as mean values ± S.D. of independent, triplicate experiments. The asterisks (*,**,***) indicate significant differences (*p* < 0.05, *p* < 0.01, *p* < 0.001,respectively)

In order to investigate whether DHRS2 inhibits cell growth through inducing cell cycle arrest, we tested cell cycle distribution in HK1-CON and HK1-DHRS2 cells. We found that over-expression of *DHRS2* significantly induced cell cycle G0/G1 phase arrest. The percentage of the G0/G1 population of cells increased to 46.0% in HK1-DHRS2 cells compared to the control (30.5%). In contrast, *DHRS2* silencing decreased the G0/G1 distribution of cells (Fig. 4a).

Cyclin D1-Cyclin-dependent kinase 4/6 (CDK4/6)-pRB axis acts as a crucial regulator of cell G1/S transition. When phosphorylated by cyclinD1-CDK4/6 complex, pRB releases numbers of transcription factors to initiate the gene transcriptional programs necessary for S phase progression. As expected, *DHRS2* overexpression decreased the expressions of cyclin D1 and CDK4 as well as phosphorylation level of RB in HK1 cells (Fig. 4b). Conversely, depletion of DHRS2 activated the CDK4-pRB axis in C666–1 cells, although the change of cyclin D1 expression was not so evident (Fig. 4c).

Considering that DHRS2 is essentially an enzyme involved in lipid metabolism [46–48], the corresponding metabolic alteration may contribute to its anti-proliferative function. To identify the proximal metabolite changes linked to *DHRS2* overexpression, we performed lipid metabolite profiling using gas chromatography coupled with mass spectrometry (GC/MS). Among the 37 lipid metabolites (Additional file 2: Table S1), lauric acid, palmitic acid, palmitoieic acid, stearic acid, elaidic acid, oleic acid, linoleic acid and arachidic acid are the most abundant fatty acids in HK1-CON and HK1-DHRS2 cells. We found that only the concentrations of oleic acid (OA) and elaidic acid (EA) were significantly decreased in HK1-DHRS2 cells in comparison to that of the control (Fig. 4d). Furthermore, MTS cell proliferation assay showed that both OA and EA partially reversed DHRS2-induced cell growth inhibition (Fig. 4e-f). Moreover, the CDK4-pRB axis was reactivated by addition either of OA or EA (Fig. 4g).

Overall, these findings support that DHRS2 may affect lipid homeostasis to reduce OA and EA production, which leads to cell cycle G0/G1 arrest and inhibition of cell proliferation in NPC cells.

### Effects of TCN on cell growth in NPC cells

**Fig. 5:**
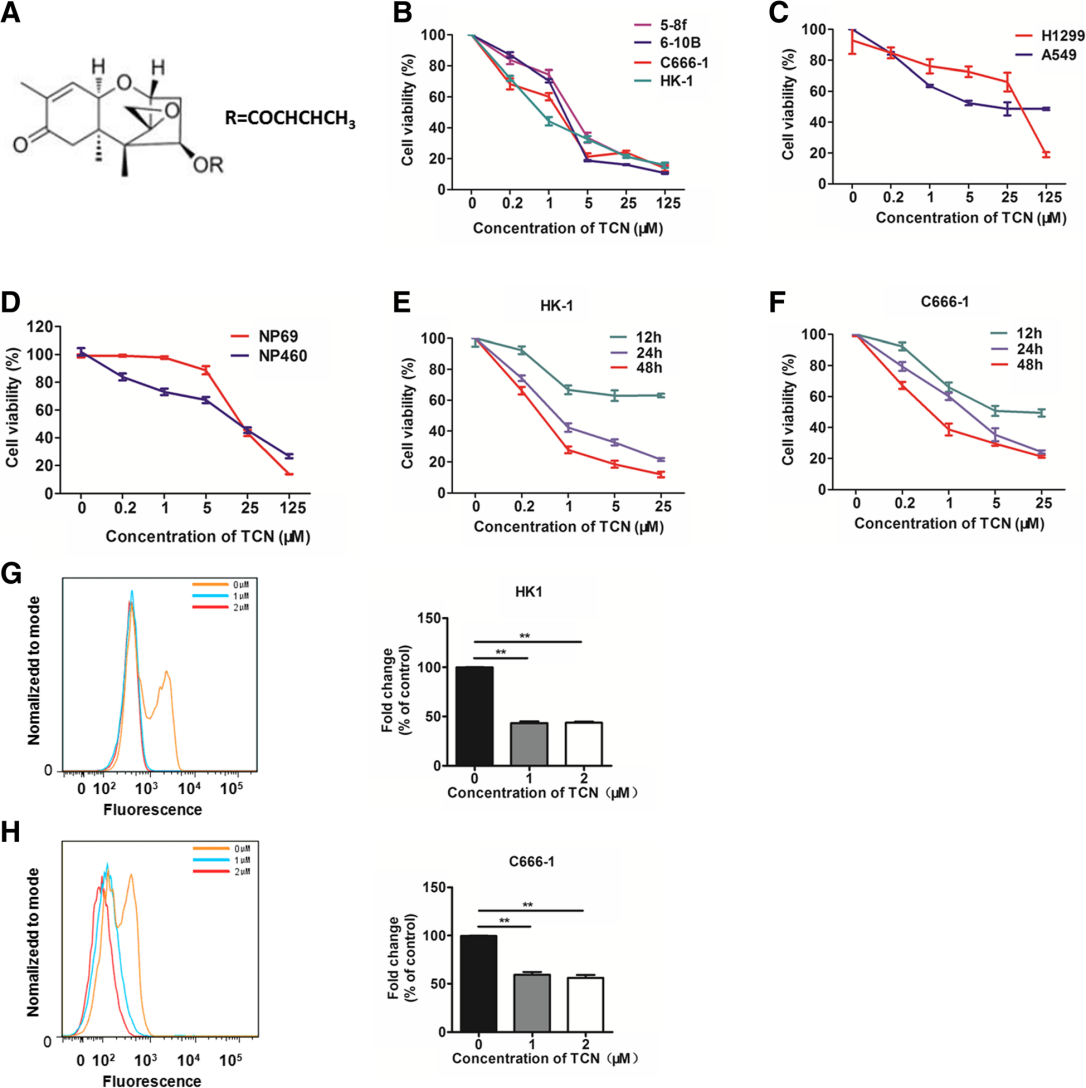
Effects of TCN on cell growth in vitro. **a** The structure of TCN. Cell growth of NPC (**b**), lung carcinoma (**c**) and immortalized nasopharyngeal epithelial cells (**d**) upon different doses of TCN treatment was analyzed by MTS assay. **e**-**f** TCN inhibits cell growth of HK1 and C666–1 cells in a time- and dose- dependent manner. Fluorescence intensity of BrdU-positive population in HK1 (**g**) and C666–1 (**h**) cells upon different doses of TCN treatment was measured by flow cytometry

In order to assess the anti-tumor activity of TCN, we performed MTS assay at the concentration of 0,0.2,1,5,25,125 μM of TCN and observed that NPC cells were more sensitive to TCN treatment (Fig. 5a-c). Moreover, TCN selectively inhibited NPC cells compared to the immortalized nasopharyngeal epithelial NP69 and NP460 cells (Fig. 5b, d, Additional file 3: Table S2). In addition, TCN treatment markedly decreased cell viability in a time-dependent manner in HK1 (Fig. 5e) and C666–1 cells (Fig. 5f). Furthermore, using BrdU staining, we measured the fluorescent intensity by flow cytometry to reflect the proliferative ability of cells. The results showed that TCN treatment at different doses resulted in significant decrease in the proliferation of NPC cells (Fig. 5g-h). These findings clearly demonstrate that TCN treatment effectively impede cell growth of NPC cells.

### DHRS2 mediates growth inhibition of NPC cells induced by TCN

**Fig. 6:**
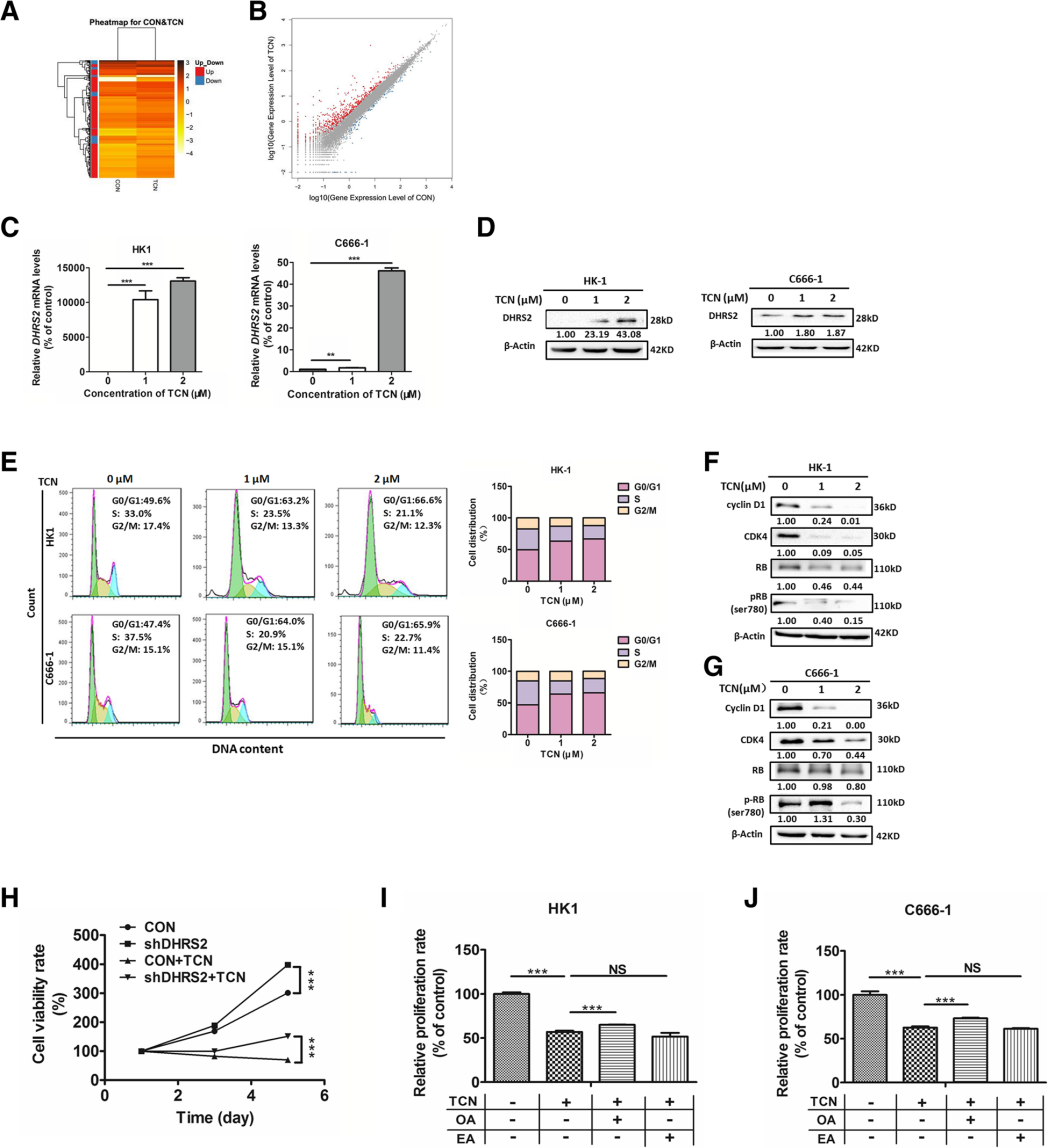
DHRS2 mediates growth inhibition of NPC cells induced by TCN. **a** Heatmap of the transcriptome changes upon TCN treatment (TCN) in comparison with the DMSO control (CON) in HK1 cells. **b** Scatter plots of all expressed genes in TCN treated group (TCN) compared with the control (CON). Red color means up-regulated gene, blue means down-regulated gene, and gray means non-regulated gene. The ‘regulated gene’ is defined as that with FDR ≤0.001 and abs(log_2_(Y/X)) ≥ 1. The mRNA (**c**) and protein (**d**) levels of DHRS2 in HK1 and C666–1 cells with different doses of TCN treatment. **e** The cell distribution was determined by flow cytometry in HK1 and C666–1 cells with different doses of TCN treatment. The protein levels of cyclinD1, CDK4, RB and pRB (Ser780) were detected by western blot assay in HK1 (**f**) and C666–1 cells (**g**) with different doses of TCN treatment. β-actin was used as a loading control. **h** Cell proliferation rate of each designated group was analyzed by MTS assay in C666–1-CON or C666–1-shDHRS2 cells in the absence or presence of 0.5uM TCN. Cell proliferation rate of each designated group was analyzed in HK1 (**i**) and C666–1 (**j**) cells. Cells were treated in the absence or presence of 0.5uM TCN, 62.5uM OA or 62.5uM EA for 72 h as designated in each group and applied for MTS assay. Data are shown as mean values ± S.D. of independent, triplicate experiments. The asterisks (**,***) indicate significant differences (*p* < 0.01, *p* < 0.001,respectively). NS, no significance

To identify the expression of genes responsible for the observed anti-tumor effect of TCN, we analyzed the transcriptome of HK1 cells in the absence or presence of TCN treatment using a RNA sequencing (RNA-seq) approach. The differentially expressed genes (DEGs) analysis showed that 494 genes were up-regulated and 107 genes were down-regulated in TCN-treated group compared with the control (Fig. 6a-b). KEGG pathway enrichment analysis illustrated that of these DEGs, 143 genes were annotated and enriched in “Metabolism”, including lipid, amino acids, nucleotide and carbohydrate metabolism (Additional file 4: Figure S2). Notably, *DHRS2* represented the most up-regulated gene involved in metabolic pathway upon TCN treatment. Based on the above RNA-seq data, we further confirmed that after TCN treatment, both mRNA and protein levels of DHRS2 were remarkably augmented in a dose-dependent manner in HK1 and C666–1 cells (Fig. 6c-d).

We next tested whether TCN suppresses the proliferation of NPC cells through affecting the cell-cycle progression. The percentage of the G0/G1 population of cells after TCN treatment increased in a dose-dependent manner relative to the untreated groups in HK1 and C666–1 (Fig. 6e). In accordance with this, the cyclin D1-CDK4-pRB axis was suppressed dose-dependently upon TCN treatment (Fig. 6f-g).

Furthermore, we investigated whether DHRS2 mediated the inhibitory effect of TCN on NPC cell growth. MTS assay was performed and we found that cell proliferation in C666–1 shDHRS2 cells was substantially propelled as compared with C666–1 CON cells. TNC treatment effectively suppressed cell growth in C666–1 CON cells, however, knockdown of DHRS2 reversed the anti-proliferative effect of TCN (Fig. 6h).

Given that DHRS2 impeded OA and EA production to induce cell cycle arrest, we further assessed whether TCN-induced cell growth inhibition could be rescued by the addition of unsaturated fatty acids in NPC cells. We observed that upon TCN treatment, the growth of NPC cells was substantially decreased relative to the untreated control. When cells were incubated in the media containing 0.125 mM OA in the presence of TCN, the growth inhibition induced by TCN was partially reversed (Fig. 6i-j). Whereas, addition of EA in the presence of TCN did not affect the cell proliferation in NPC cells (Fig. 6i-j). Overall, these observations suggest that the up-regulation of DHRS2 mediates the inhibitory effect on NPC cell growth by TCN treatment.

### TCN up-regulates DHRS2 to inhibit NPC growth in vivo

**Fig. 7:**
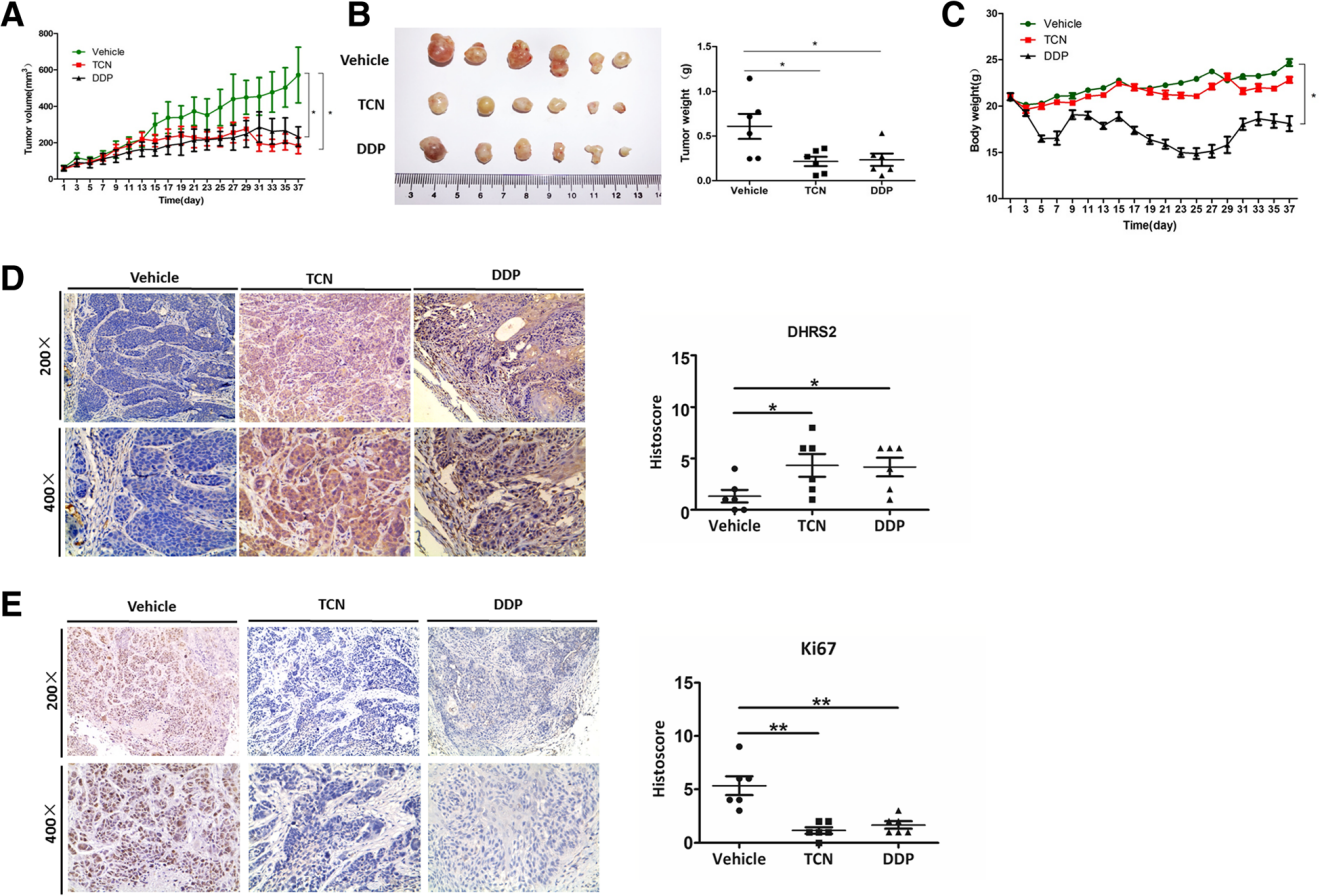
TCN up-regulates DHRS2 to inhibit NPC growth in vivo. **a** Effect of TCN on HK1 NPC cell xenograft in athymic BALB/c nude mice. Nude mice bearing HK1 cells were randomly separated into 3 groups (*n* = 6) and treated with corn oil (vehicle), TCN (1 mg/kg) or DDP (5 mg/kg) every other day for 5 weeks. Tumor volume was examined every other day and shown in the graph. **b** At the end of the experiment, the mice were sacrificed and the tumors were separated. Tumor mass of each group was weighed and shown in the graph. **c** During the experiment, body weight of the mice in each group was monitored and shown in the graph. **d**-**e** Images of tumor sections in each group stained with indicated antibodies. Antibody staining is in brown and nuclear counter staining is in blue. Scatter diagram shows Histoscore for the indicated antibody staining in tumor samples

To interrogate the inhibitory effect of TCN on NPC in vivo, we performed xenograft experiment using HK1 cells, which is a representative of the in vitro studies. Cisplatin (DDP) is a clinical first-line drug for NPC chemotherapy. In the xenograft experiment, we used it as a positive control. Treatment with TCN in HK1 xenografts at a dose of 1 mg/kg resulted in a significant inhibition of tumor load by 3-fold compared to the vehicle group. Meanwhile, DDP treatment achieved a similar tumor suppression effect as that in TCN group, while at a much higher dose of 5 mg/kg (Fig. 7a-b). In addition, DDP group showed a more severe side effect with obvious loss of weight relative to TCN or vehicle group (Fig. 7c). Moreover, we assessed DHRS2 by immunohistochemistry (IHC) on the xenograft NPC tumor samples of each group. A scoring scale was applied to quantify DHRS2 staining, which combines the staining intensity with the percentage of positive cells (Histoscore). We found that DHRS2 staining was markedly enhanced in TCN group compared to the vehicle control. Likewise, DDP treatment up-regulated DHRS2 expression in vivo as well (Fig. 7d). In contrast, the staining of Ki67, a cell proliferative marker, was remarkably suppressed both in TCN and DDP groups relative to the control (Fig. 7e). These results indicate that TCN may up-regulates DHRS2 to inhibit tumor growth in vivo.

## Discussion

A growing body of evidence displays the close connection between cancer and lipid metabolism. Cellular rapid proliferation, a common characteristic of cancers, relies on FAs for membrane formation, synthesis of signaling molecules and energy storage [49].

In this paper, we first confirmed that DHRS2 expression was much lower in NPC cells relative to the immortalized nasopharyngeal epithelial cells. Using EdU and colony formation assays in addition to MTS assay, we further demonstrated that DHRS2 inhibits cell growth in NPC cells.

*DHRS2* gene codes for an enzyme that is a member of SDR superfamily. As NAD(P)(H)-dependent dehydrogenases/reductases, SDR enzymes act on a heterogeneous set of lipid-related substrates, including fatty acids, steroids and retinoids [50]. Sequence alignment of DHRS2 shows significant homology to SDR enzymes, with maximum similarity at the N-terminal part, corresponding to the NAD/NADP cofactor-binding domain. Moreover, conservations at Yl85 and K189 are also observed, which are involved in the enzymatic catalytic function [27]. Although the document referring to metabolic function of DHRS2 is limited, it should play considerable roles in lipid metabolism. Accordingly, we performed a lipid metabolite profiling analysis on NPC cells with over-expression of *DHRS2* and the control, to identify critical FA metabolite alteration that may contribute to the role of DHRS2 in NPC progression. We found the levels of two unsaturated FAs, OA and EA were significantly reduced by *DHRS2* overexpression. Addition of either of them was capable of promoting cell-cycle progression to rescue cell growth inhibited by DHRS2.

In addition to its metabolic function, DHRS2 also reportedly mediated the stability of p53 through interaction with MDM2. Nevertheless, we observed no obvious change on MDM2 expression while a sharp decrease on p53 level in HK1-DHRS2 cells compared to the control. Reversely, knockdown of DHRS2 augmented p53 expression (Additional file 5: Figure S3). Thereby, in this scenario, MDM2/p53 axis may be not involved in the function of DHRS2 to induce cell cycle arrest.

Owing to inherently abundant structural diversity, natural compounds provide unique source for discovering innovative drugs [51]. TCN, a natural sesquiterpenoid compound isolated from an endophytic fungus, exerts significant anti-tumor activity selectively in NPC cells relative to immortalized nasopharyngeal epithelial cells or lung carcinoma cells. Using an RNA-seq approach, we identified *DHRS2* is the most up-regulated metabolic- associated gene upon TCN treatment and further confirmed this through in vitro and in vivo assays. Moreover, we found that addition of OA effectively reverses the inhibitory effect of TCN. *DHRS2* was identified as a downstream gene of Homeobox A13 (HOXA13), which directly down-regulated DHRS2 to increase MDM2 [25]. *DHRS2* was also reported to be a c-Myb target gene [48]. However, although TCN treatment actually augmented the transcriptional activity of *DHRS2* promoter region, it failed to increase transcription factor c-Myb levels (Additional file 6: Figure S4). Hence, c-Myb might not mediate the up-regulation of DHRS2 upon TCN treatment.

## Conclusion

Our report supports the notion that DHRS2 may mediate the anti-proliferative function of TCN in NPC cells at least partially through limiting supplies of unsaturated FAs. Notably, TCN retards tumor growth more effectively in vivo at a lower dose and less side effects, compared to that with DDP treatment. Therefore, we propose that activating DHRS2 to reprogram lipid homeostasis may be a target for intervention against NPC. TCN could be exploited for therapeutic gain by targeting DHRS2 and it may also be developed as a tool to enhance understanding the biological function of DHRS2.

## Additional files

Additional file 1: **Figure S1.** (A) The protein levels of DHRS2 in immortalized nasopharyngeal epithelial cells and NPC cells. (B) The protein levels of DHRS2 in C666–1 cells transfected with control shRNA or *DHRS2* shRNAs (1#, 2# and 3#). (TIF 1526 kb)

Additional file 2: **Table S1.** 37 Standard analytes applied for the GC/MS analysis (XLSX 9 kb)

Additional file 3: **Table S2.** IC50 values of the inhibitory effect of TCN on tumor cells and immortalized normal cells. (DOCX 12 kb)

Additional file 4: **Figure S2.** KEGG Pathway enrichment analysis of all expressed genes in TCN treated group (TCN) compared with the control (CON). In TCN vs CON, the DEGs were enriched in “cellular processes”, “environmental information processing”, “genetic information processing”, “human diseases”, “metabolism” and “organismal systems”. (TIF 2016 kb)

Additional file 5: **Figure S3.** The effect of overexpression or knockdown of DHRS2 on MDM2-p53 axis in NPC cells. The protein levels of MDM2 and p53 were detected by western blot assay. (TIF 392 kb)

Additional file 6: **Figure S4.** TCN treatment up-regulates *DHRS2* transcription. The effect on *DHRS2* promoter activity upon TCN treatment in HK1 (A) and C666–1 (B) cells. After transfection with DHRS2-luc followed by treatment with TCN (1 μM) for 24 h, firefly luciferase activity reflecting *DHRS2* promoter activity was measured and normalized to Renilla luciferase activity. (C) The effect of TCN on c-Myb protein levels in HK1 and C666–1 cells. β-actin was used as a loading control. (TIF 1766 kb)

## Data Availability

Data sharing not applicable to this article as no datasets were generated or analysed during the current study.

## Abbreviations

DDP: Cisplatin
DEGs: Differentially expressed genes
DHRS2: Dehydrogenase/reductase member 2
EA: Elaidic acid
EdU: 5-ethynyl-2′- deoxyuridine
FAs: Fatty acids
FBS: Fetal bovine serum
GC/MS: Gas chromatography coupled with mass spectrometry
GO: Gene Ontology
IHC: Immunohistochemistry
IOD: Integrated Optical Density
KEGG: Kyoto Encyclopedia of Genes and Genomes
MTS: 3-(4,5-dimethylthiazol-2-yl)-5- (3-carboxymethoxyphenyl)-2-(4-sulfophenyl)- 2H-tetrazolium
NPC: Nasopharyngeal carcinoma
OA: Oleic acid
RNA-seq: RNA sequencing
S1P: Sphingosine-1-phosphate
SDR: Short-chain alcohol dehydrogenase/reductase
SFM: Serum-free medium
shRNA: Short hairpin RNA
ssCir DNAs: Single strand circle DNAs
TCN: Trichothecin
