# Supplementary figures and images for: DHRS2 mediates cell growth inhibition induced by Trichothecin in nasopharyngeal carcinoma
Source: J Exp Clin Cancer Res. 2019 Jul 10;38:300. doi: 10.1186/s13046-019-1301-1 (PMC6617617; doi:10.1186/s13046-019-1301-1)

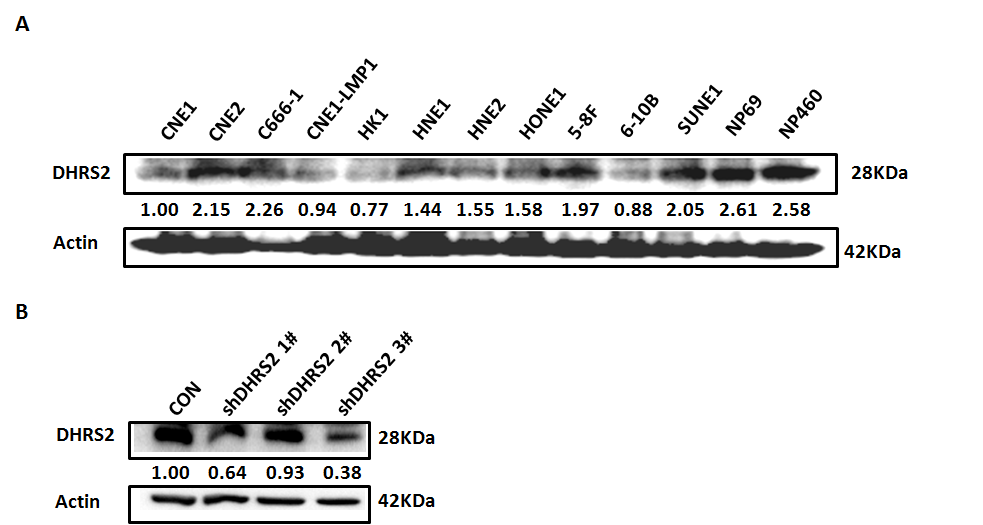

Supplement: Supplementary file 1 — Figure S1. (A) The protein levels of DHRS2 in immortalized nasopharyngeal epithelial cells and NPC cells. (B) The protein levels of DHRS2 in C666–1 cells transfected with control shRNA or DHRS2 shRNAs (1#, 2# and 3#). (TIF 1526 kb) [file 13046_2019_1301_MOESM1_ESM.tif]

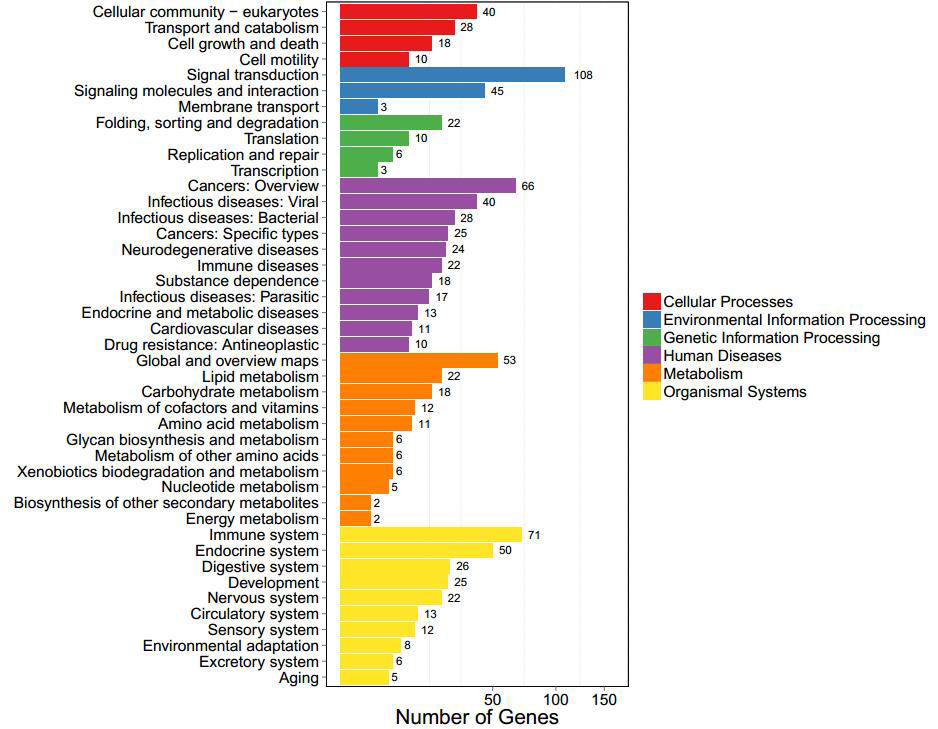

Supplement: Supplementary file 4 — Figure S2. KEGG Pathway enrichment analysis of all expressed genes in TCN treated group (TCN) compared with the control (CON). In TCN vs CON, the DEGs were enriched in “cellular processes”, “environmental information processing”, “genetic information processing”, “human diseases”, “metabolism” and “organismal systems”. (TIF 2016 kb) [file 13046_2019_1301_MOESM4_ESM.tif]

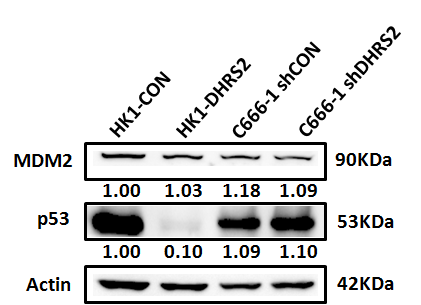

Supplement: Supplementary file 5 — Figure S3. The effect of overexpression or knockdown of DHRS2 on MDM2-p53 axis in NPC cells. The protein levels of MDM2 and p53 were detected by western blot assay. (TIF 392 kb) [file 13046_2019_1301_MOESM5_ESM.tif]

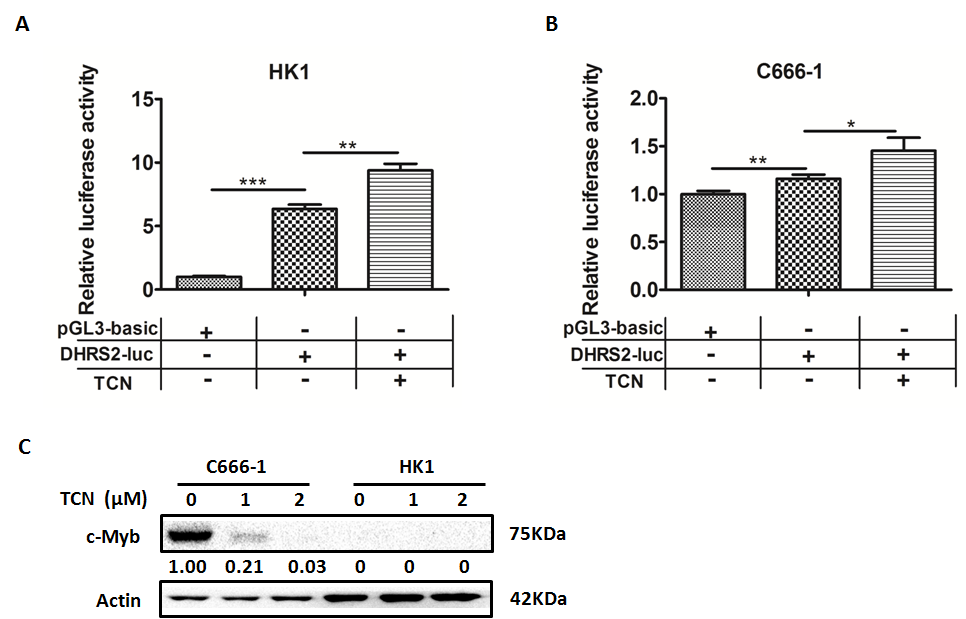

Supplement: Supplementary file 6 — Figure S4. TCN treatment up-regulates DHRS2 transcription. The effect on DHRS2 promoter activity upon TCN treatment in HK1 (A) and C666–1 (B) cells. After transfection with DHRS2-luc followed by treatment with TCN (1 μM) for 24 h, firefly luciferase activity reflecting DHRS2 promoter activity was measured and normalized to Renilla luciferase activity. (C) The effect of TCN on c-Myb protein levels in HK1 and C666–1 cells. β-actin was used as a loading control. (TIF 1766 kb) [file 13046_2019_1301_MOESM6_ESM.tif]
