# Supplementary material for: DHRS2 mediates cell growth inhibition induced by Trichothecin in nasopharyngeal carcinoma
Source: J Exp Clin Cancer Res. 2019 Jul 10;38:300. doi: 10.1186/s13046-019-1301-1 (PMC6617617; doi:10.1186/s13046-019-1301-1)
Supplement: Supplementary file 3 — Table S2. IC50 values of the inhibitory effect of TCN on tumor cells and immortalized normal cells. (DOCX 12 kb) [file 13046_2019_1301_MOESM3_ESM.docx]

Table 2 IC50 values of the inhibitory effect of TCN on tumor cells and immortalized normal cells

|  |  |  |  |
| --- | --- | --- | --- |
|  | Cell line | IC50（uM） |  |
|  | HK-1 | 1.02 |  |
|  | C666-1 | 1.19 |  |
|  | 5-8F | 4.53 |  |
|  | 6-10B | 3.35 |  |
|  | NP69 | 21.91 |  |
|  | NP460 | 15.66 |  |
|  | H1299 | 29.83 |  |
|  | A549 | 27.33 |  |
|  |  |  |  |
